# Predicting United States Medical Licensing Examination Step 2 Clinical Knowledge Scores from Previous Academic Performance Measures within a Longitudinal Interleaved Curriculum

**DOI:** 10.7759/cureus.18143

**Published:** 2021-09-20

**Authors:** Rachel A Kracaw, Wynona Dizon, Sabrina Antonio, Edward Simanton

**Affiliations:** 1 Medical Education, University of Nevada, Las Vegas School of Medicine, Las Vegas, USA

**Keywords:** prediction model, pass/fail usmle step 1, usmle step 2 correlations, usmle step 2, predicting usmle step 2

## Abstract

Background

United States Medical Licensing Examination (USMLE) Step 1 is a common metric looked at by residency programs to determine invitations for candidates to interview. However, USMLE Step 2 Clinical Knowledge (CK) has also been an important factor for selecting applicants to interview and plays a significant role during applicant selection. This study aims to identify academic performance measures that correlate with USMLE Step 2 CK scores and to develop a model to predict USMLE Step 2 CK scores using previous academic measures from the first two cohorts in the longitudinal interleaved clerkship (LInC) at the Kirk Kerkorian School of Medicine at the University of Nevada, Las Vegas (KSOM).

Setting

The KSOM is a newly accredited US allopathic medical school that accepted its first class in 2017. At KSOM, a LInC model is used in the primary clinical year. In this model, rotations are two weeks in duration before moving on to the next specialty. Students complete the National Board of Medical Examiners (NBME) subject examinations in all six specialties in one week at the midpoint and the end of the LInC. Students who passed an exam at the midpoint can opt out of that exam at the end as the higher of the two exam scores is recorded. However, most students choose to take all the exams again to improve their scores and prepare for USMLE Step 2 CK.

Methodology

Academic performance measures were gathered from the class of 2021 and 2022 (n = 101) including undergraduate grade point average (GPA), undergraduate science GPA, medical college admission test score, USMLE Step 1 score, NBME clinical subject exam scores, and USMLE Step 2 CK scores. Pearson correlations were run between the performance variables and USMLE Step 2 CK scores to measure influence variables individually, then a regression model measured impacts of variables together.

Results

All variables except undergraduate science GPA significantly correlated with USMLE Step 2 CK score. USMLE Step 1 had the strongest correlation (r = 0.752, p < 0.001). The regression model had an R of 0.859 with the internal medicine subject exam showing the highest beta coefficient (0.327, p < 0.001).

Conclusions

This study determined that USMLE Step 2 CK scores can be effectively predicted using available performance measures. With USMLE Step 1 becoming pass/fail in January 2022, the importance of USMLE Step 2 CK as a screening tool in the residency application process will likely increase. This study was conducted within a LInC curriculum and may have limited value in the prediction of scores within other clinical year curricula.

## Introduction

The United States Medical Licensing Examination (USMLE) has three exams consisting of Step 1, Step 2 Clinical Knowledge (CK), and Step 3. USMLE Step 1 tests students’ knowledge of science and preclinical medicine, whereas USMLE Step 2 CK focuses more on clinical medicine. Traditionally, USMLE Step 1 has been the most impactful measure looked at by residency programs to determine invitations for candidates to interview according to the National Resident Matching Program (NRMP) data in 2020 [1]. USMLE Step 1 scores have been correlated with resident performance and ability to pass medical licensing board examinations [2-4]. USMLE Step 2 CK also correlates with resident performance and ability to pass medical licensing board examinations, but according to NRMP data, it has not been as important as a measure, compared to USMLE Step 1, to select residency candidates [1-4].

Many studies have attempted to predict student performance on USMLE Step 1 [4-7]. However, given that USMLE Step 1 will become a pass/fail exam in January 2022, the necessity is now to predict USMLE Step 2 CK scores given its increasing importance in the residency application process [5,8,9]. To date, there are limited studies available to predict USMLE Step 2 CK [4,10,11].

Several factors have been shown to correlate with USMLE Step 1 performance. Preparation methods, study resources, science exposure before medical school, medical college admission test (MCAT), undergraduate grade point average (GPA), National Board of Medical Examiners (NBME) clinical subject examinations, National Institute of Health funding, and socioeconomic status all play a role in the performance [3-7,12-18]. However, limited studies exist that determine whether these performance measures also correlate with USMLE Step 2 CK scores. Monteiro et al. demonstrated that USMLE Step 1 score, preclinical mean exam score, NBME subject exams (obstetrics and gynecology, family medicine, pediatrics, internal medicine, and surgery) all correlated with USMLE Step 2 CK performance [10]. Ogunyemi et al. determined that USMLE Step 1, the NBME obstetrics and gynecology subject exam, and MCAT were the strongest predictors of USMLE Step 2 CK [19]. Other studies support a strong correlation between USMLE Step 1 and MCAT with USMLE Step 2 CK performance [7,12]. Interestingly, correlations also exist between medical student clinical clerkship performance and USMLE Step 2 CK scores [2,3,17]. However, previous studies do not combine variables into a predictive model to help estimate USMLE Step 2 CK scores of students.

This study aims to identify academic performance measures that correlate with USMLE Step 2 CK scores and to develop a model to predict USMLE Step 2 CK scores using previous academic measures. This study is based on data from the first two cohorts at Kirk Kerkorian School of Medicine at the University of Nevada, Las Vegas (KSOM). The academic performance measures under evaluation for predictivity with USMLE Step 2 CK scores are total undergraduate GPA, undergraduate science GPA, MCAT score, USMLE Step 1 score, and scores for each NBME subject exam (medicine, surgery, obstetrics and gynecology, pediatrics, psychology, and family medicine).

## Materials and methods

The KSOM is a newly accredited US allopathic medical school that accepted its first class in 2017. At the KSOM, a Longitudinal Interleaved Clerkship (LInC) model is used in the primary clinical year. In this model, specialty rotations are two weeks in duration before moving on to the next specialty. This differs from most Longitudinal Integrated Clerkships (LIC) which traditionally use half-day experiences in each specialty. Students complete four rotations (total of eight weeks) in surgery and internal medicine. All other specialties have three rotations (a total of six weeks). Two elective rotations are also included in the LInC to allow for career exploration. Each student’s schedule is unique and rotations are scheduled to ensure that every student has a minimum of one rotation in each specialty in the first half of the year and again in the second half. Six weeks of independent study are provided at the end of the LInC that can be used for either exam preparation, completion of required clinical experiences missed during regularly scheduled rotations, or optional clinical experiences. Students complete NBME subject exams in all six specialties in one week at the midpoint of the LInC. At the end of the LInC, all exams are offered again over six consecutive days (one exam per day). Students who passed an exam at the midpoint can opt out of that exam at the end but most students choose to take all the exams again to improve their scores and prepare for USMLE Step 2 CK. The highest score of the two attempts is reported for the student’s subject exam grade in each specialty. Students are encouraged to study the specialty of their current rotation but also spend time studying broadly.

At the time of this study, two cohorts at the KSOM had completed the LInC and taken Step 2 CK. The class of 2021 (n = 50) and 2022 (n = 51) provided a total of 101 subjects. Deidentified academic performance measures were gathered from existing institutional databases in accordance with an approved IRB protocol. Premedical performance measures included total undergraduate GPA, undergraduate science GPA, and MCAT score. Preclinical performance variables were mean exam performance during the basic science phase and USMLE Step 1 score. Clinical phase variables included scores for each NBME clinical subject exam (family medicine, medicine, obstetrics and gynecology, pediatrics, psychology, and surgery).

Pearson correlation coefficients were calculated between each of the variables and the USMLE Step 2 CK score. This determined relationships between each of the performance measures and USMLE Step 2 CK scores. Then a regression was employed (Method = Enter) using the same variables to form a prediction model.

## Results

Pearson correlations for all of the academic performance measures with USMLE Step 2 CK scores can be found in Table 1.

**Table 1:** Results of academic performance measures’ correlation with USMLE Step 2 CK scores. * Statistically significant with an alpha of 0.05. USMLE: United States Medical Licensing Examination; CK: Clinical Knowledge; MCAT: Medical College Admission Test; GPA: grade point average

Academic performance measure	R-value	P-value
Family Medicine Subject Exam	0.627	<0.001*
Medicine Subject Exam	0.745	<0.001*
Obstetrics and Gynecology Subject Exam	0.616	<0.001*
Pediatric Subject Exam	0.672	<0.001*
Psychology Subject Exam	0.530	<0.001*
Surgery Subject Exam	0.617	<0.001*
USMLE Step 1	0.752	<0.001*
Basic Sciences Exam Mean	0.604	<0.001*
MCAT	0.276	0.005*
Undergraduate GPA	0.233	0.019*
Undergraduate Science GPA	0.196	0.050

The variables most correlated included USMLE Step 1 (r = 0.752, p < 0.001), the medicine NBME subject exam (r = 0.745, p < 0.001), and the pediatric NBME subject exam. The least correlated variables included MCAT (r = 0.276, p = 0.005) and undergraduate GPA (r = 0.233, p = 0.019). The only variable not significantly correlated with USMLE Step 2 CK was undergraduate science GPA (r = 0.196, p = 0.050).

For the regression model predicting USMLE Step 2 CK scores, the Pearson coefficient was reported as an R-value of 0.859 (p < 0.001). The standardized and unstandardized coefficients along with their respective p-values are shown in Table 2.

**Table 2:** Standardized and unstandardized coefficients for the predictive model to predict USMLE Step 2 CK scores. * Statistically significant with an alpha of 0.05. USMLE: United States Medical Licensing Examination; CK: Clinical Knowledge MCAT: Medical College Admission Test; GPA: grade point average

Academic performance measure	Standardized coefficient	Standard error	Unstandardized coefficient (beta)	P-value
Family Medicine	0.349	0.219	0.134	0.114
Medicine	0.634	0.175	0.327	<0.001*
Obstetrics and Gynecology	0.086	0.203	0.036	0.672
Pediatrics	0.355	0.179	0.171	0.051
Psychiatry	0.077	0.235	0.023	0.744
Surgery	-0.117	0.170	-0.060	0.493
Basic Sciences Exam mean	32.721	24.083	0.114	0.178
USMLE Step 1	0.281	0.084	0.321	0.001*
MCAT	-0.168	0.181	-0.060	0.356
Undergraduate GPA	17.549	8.388	0.390	0.039*
Undergraduate Science GPA	-16.038	6.847	-0.432	0.021*
Constant	123.897	85.680	-	0.152

The variables with significant beta coefficients included NBME medicine subject exam (b = 0.327, p < 0.001), USMLE Step 1 score (b = 0.321, p = 0.001), undergraduate science GPA (b = -0432, p = 0.021), and undergraduate science GPA (b = 0.390, p = 0.039).

**Figure 1:**
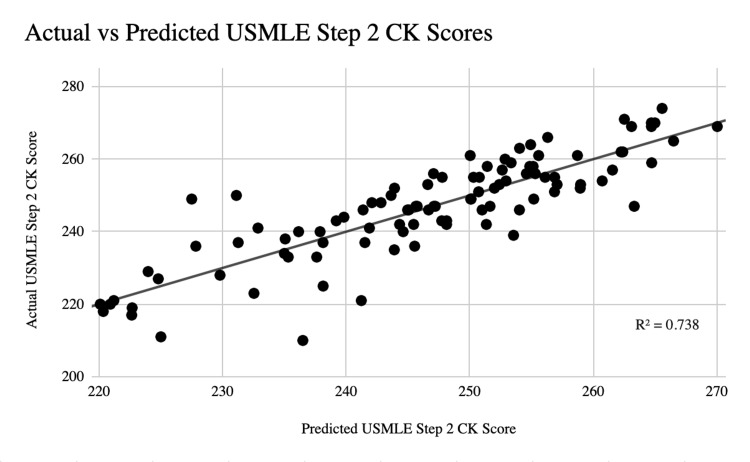
Actual versus predicted USMLE Step 2 CK scores. USMLE: United States Medical Licensing Examination; CK: Clinical Knowledge

The percentage error and difference for the predictive regression model are shown in Table 3, and Figure 1 shows a graph of the actual versus the predicted USMLE Step 2 CK score.

**Table 3:** Averages, minimums, maximums, and standard deviations for the prediction model percentage error and the difference between actual versus predicted. USMLE: United States Medical Licensing Examination; CK: Clinical Knowledge

Statistic	Average	Minimum	Maximum	Standard deviation
Difference between actual versus predicted USMLE Step 2 CK	0	-26	21	2.100
Percentage error	2.199%	0%	12.617%	7.240%

## Discussion

This study determined that USMLE Step 2 CK scores can be effectively predicted using available performance measures. The variables with the strongest correlations were USMLE Step 1, the NBME medicine subject examination, and the NBME pediatrics subject examination. The lowest correlated measures were MCAT scores and undergraduate GPA. Undergraduate science GPA was the only variable that was not significantly correlated with USMLE Step 2 CK. All but one variable was significantly correlated, which shows these measures are predictive. Comparable trends have been found in studies that examine similar academic performance measures correlated with USMLE Step 1, which was expected, given that USMLE Step 1 and USMLE Step 2 CK also correlate well [3-7,10,12-19]. However, these correlations could also be explained by students’ academic aptitudes, as demonstrated previously [17]. A similar correlation showed that poor performance on standardized exams before and early in medical school is associated with poor performance on other exams later in school and beyond [3].

For the predictive model, the average percentage error and the r-value for the Pearson coefficient model both indicate that this model is highly predictive of student performance on USMLE Step 2 CK. Therefore, using previous academic performance measures can help determine performance on USMLE Step 2 CK. It is important to start predicting USMLE Step 2 CK scores given its rising importance in the residency application process [8,9]. With the introduction of a pass/fail USMLE Step 1 beginning January 2022, USMLE Step 2 CK will likely become more widely used during the residency application process [8,9]. Before this transition to pass/fail, USMLE Step 1 was used heavily as a cutoff for residency program interview invitations [1]. Because of this correlation, residency programs are projected to use USMLE Step 2 CK as they have used USMLE Step 1 in the past [8,9]. This exam will likely become the new deciding factor when determining to interview residency candidates due to USMLE Step 1 becoming pass/fail and the fact that USMLE Step 2 CK focuses more on clinical knowledge which is more applicable to residency performance [8,9].

Several limitations exist in this study. First, all participants were students from the charter and second class at KSOM. Being a new school could have influenced test scores and other academic measures because the curriculum is new and less developed compared to more established institutions. Second, the students involved in the study participated in a LInC model for their third year of medical school. Recent studies have shown that a longitudinal clerkship model can increase standardized test scores compared to the traditional clerkship model [20]. This is likely because students benefit from having to continuously study all specialty subjects throughout their clerkship year rather than studying one specialty at a time. Another benefit to the LInC model is that it removes the bias from the order in which the NBME subject exams were taken because KSOM administers all these exams at once rather than sequentially after each respective rotation. Some studies show that the order in which NBME subject exams are taken can affect scores on subsequent subject exams [21]. For example, the family medicine exam contains much medicine and obstetrics and gynecology content. Thus, if the family medicine exam is completed after having taken and completing medicine and obstetrics and gynecology, students will feel more prepared [21]. The LInC model also allows the NBME subject exams to be taken twice with the highest of the two scores being reported, thus, giving students more exposure to these exams, which may influence the model’s accuracy for other programs. Taking all six NBME subject exams in a one-week period could affect the predictive model and the actual USMLE Step 2 CK scores because students are either fatigued from so many exams or they have the opportunity to build their mental endurance to be able to sit for the USMLE Step 2 CK exam. Third, this study used USMLE Step 1 as a preclinical variable in the predictive model. As previously stated, USMLE Step 1 will become a pass/fail exam with a non-numeric score in January 2022. It cannot be used as a variable in future predictive models. Lastly, the number of students included in the study was small and the correlations could likely be strengthened if more students were included.

Interestingly, performance in medical school is a much better predictor of USMLE results than pre-admission variables [11]. In future studies, it would be beneficial to include more pre-clinical and clinical predictors rather than pre-admission variables. Kleshinski et al. found an unconventional correlation between age and USMLE Step 2 CK performance that has not been demonstrated elsewhere in the literature [7]. This study did not include student age in the analysis but could in future iterations. Lastly, a predictive model exists for USMLE Step 1 using measures such as study resources, study methods, length of study, financial need, and ethnicity, which could also be useful in future studies [5].

## Conclusions

This study correlated academic performance measures with USMLE Step 2 CK scores to help predict students’ performance on USMLE Step 2 CK. In the future, adding in other measures such as age, financial need, and ethnicity could help improve the predictive model. For future iterations, this model could be more realistic if it did not include USMLE Step 1 scores, given the transition to pass/fail scoring system.
